# Optimizing the Interaction System for Treadmill Video Games Using a Smartphone’s Front Camera

**DOI:** 10.3390/s26010020

**Published:** 2025-12-19

**Authors:** Micaela Yanet Martin, Carlos Marín-Lora, María Beatriz Villar-López, Miguel Chover

**Affiliations:** 1 Institute of New Imaging Technologies, Universitat Jaume I, Av. Vicent Sos Baynat s/n, 12071 Castellón de la Plana, Spain; mavillar@uji.es (M.B.V.-L.);; 2 Department of Computer Science and Systems Engineering, University of Zaragoza. Escuela Universitaria Politécnica de Teruel, C/Ciudad Escolar, nº2, 44003 Teruel, Spain

**Keywords:** treadmill interaction, face tracking, mobile technology, active video games

## Abstract

This paper introduces a lightweight and accessible interaction system for treadmill-based video games, relying solely on facial tracking via a smartphone’s front camera. The system enables real-time estimation of running cadence and directional control through natural head movements, providing an immersive and hands-free gaming experience. A key contribution is the implementation of a FFT-based cadence estimation method that achieves accuracy errors below 5% using only 128 frames, enabling real-time feedback. Preliminary evaluations with 11 participants demonstrate that the FFT-based approach outperforms traditional peak detection in both accuracy and robustness across multiple running speeds. These results position the system as a practical, efficient, and scalable solution for fitness-oriented human–computer interaction, with promising implications for digital health and exergaming.

## 1. Introduction

The development of interactive systems to enhance treadmill exercise experiences has become a critical area of innovation [1,2], particularly at the intersection of wearable technology and fitness. Leveraging the wide availability and capabilities of smartphones, we aim to create advanced treadmill training systems that not only meet the growing demand for immersive and personalized training experiences, but also harness the potential of mobile technology to transform conventional exercise routines. This approach significantly increases user motivation [3,4] and redefines the way we interact with exercise equipment, making workouts more engaging and effective.

The main objective of this work is to optimize the face-tracking interaction time using smartphones for seamless integration into treadmill-based exergames, ensuring real-time rendering and responsiveness. In this paper, we will understand exergame following the definition given in [5], as “a digital game that requires players to make a (voluntary) physical effort to play and has specific objectives, rules, and a feedback mechanism”, adding the condition that the objective of the game is not the physical activity itself.

Previous research on treadmill-based gaming and interaction has explored various approaches, including wearable devices like bracelets, heart rate monitors, and smartwatches for gesture and cadence detection [6,7,8], camera-based systems such as webcams and Kinect sensors for contactless control [9,10], smartphone-based solutions using accelerometers or microphones [11,12], running-in-place VR exergames using HMDs [13], and treadmill-based locomotion interfaces designed to support omnidirectional or advanced movement capabilities [14]. However, these methods often require specialized hardware, have high computational costs, or exhibit limited responsiveness due to long analysis windows. Furthermore, previous face-tracking methods required video sequences of more than 16 s to achieve accurate cadence estimation (less than 5% error) [15], limiting their real-time interactivity. To overcome these issues, our work proposes a smartphone-only system capable of accurately estimating cadence using much shorter video segments, enabling fast, hands-free interaction without the need for additional hardware.

To simplify interaction and eliminate the need for additional devices such as smartwatches or pedometers, we have designed an optimized system that uses the front camera of a smartphone to analyze and track the user’s facial movements in real time. The smartphone remains fixed on the treadmill, becoming the only device necessary to capture and process information without the need for additional hardware.

In this context, we define “accessible” as an interaction approach that requires only a standard smartphone, without additional wearable sensors or specialized hardware, thereby reducing costs and setup requirements and making treadmill-based games available to a broader range of users. Moreover, although our method relies on the use of a smartphone, it does not constitute a hardware-specific strategy, as smartphones are widely available devices: recent data indicate that approximately 71% of the global population owns a smartphone as of 2024 [16], ensuring broad compatibility and barrier-free access for most users.

Our proposed system improves interactivity and adaptability in treadmill-based video games by enabling real-time facial tracking through a smartphone’s front camera. It addresses key limitations of previous treadmill systems, such as the need for additional hardware or complex setups, by offering a practical, low-cost solution that enhances safety, immersion, and responsiveness. The cadence estimation method, based on frequency analysis of head motion, provides a robust and efficient alternative to gesture-based or wearable-dependent systems. Combined with intuitive directional control via natural head movements, the system delivers a streamlined and hands-free interaction experience, contributing to more accessible and engaging fitness-oriented applications.

In summary, this paper is structured as follows. Section 2 reviews the state of the art in treadmill interaction systems, highlighting the limitations and opportunities in current technologies. Section 3 presents the system setup. Section 4 the methodology for developing the face-tracking interaction. Section 5 presents the experimental results, evaluates the system’s performance in terms of cadence estimation and user interaction, and discusses the implications for improving treadmill workouts. Section 6 concludes with a summary of the study’s contributions and possible future research directions.

## 2. State of the Art

The development of treadmill interaction systems has evolved significantly, with early approaches such as Ahn et al. [6] and Marin-Lora et al. [7] using bracelets or smartwatches to adjust speed and control avatars. These systems enabled dynamic interaction but faced limitations in gesture recognition and dependency on specific wearables.

Face-tracking interaction has also been explored. Lee and Lee [17] used webcams for facial and hand gesture control in games, while Ilves et al. [18] highlighted head movements and facial expressions as input to enrich gameplay. Olszewski et al. [19] presented monocular camera systems in HMDs to create real-time facial animations, though these methods required specialized hardware and were limited to static or seated contexts.

Regarding mobile interactions, Pauly [20] and Anderegg et al. [21] developed systems where avatars responded to smartphone-based movements, but these did not consider treadmill environments. Zhao et al. [22] advanced facial gesture interaction by combining head movements with touch and device sensors, yet still required the device to be held by the user, limiting natural running form. Georgiadis and Yousefi [23] focused on gesture segmentation for mobile VR but did not address treadmill running.

Other works like Hu et al. [9] and Hai et al. [10] used cameras or Kinect for treadmill-based interactions, but their reliance on external hardware reduced accessibility. Studies such as Hamada et al. [24] and Keshner et al. [25] emphasized virtual avatars’ motivational benefits, but without proposing technical solutions for treadmill control.

Research on cadence estimation includes accelerometer-based methods [11], microphone-based systems [12], and facial tracking approaches [15].

Accelerometer-based methods have served as the foundation for most cadence estimation research due to their portability and low power requirements. One of the key contributions in this area is the RRACE algorithm proposed by Karuei et al., which provides a robust real-time cadence estimation technique using accelerometer data processed through adaptive peak detection and filtering. This method demonstrated reliable cadence tracking during both walking and running, even under noisy sensor conditions [11]. Similarly, Davis et al. [26] employed a machine learning approach to estimate running speed and cadence using accelerometer data from wrist- and waist-worn devices, showing that supervised models can outperform traditional signal-processing methods in unconstrained environments.

Beyond accelerometers, alternative sensing modalities have been explored to enhance robustness and user convenience. Abdulla et al. [27] investigated the use of magnetometers for walking and running cadence estimation, leveraging the periodic magnetic fluctuations produced by limb motion. Their study demonstrated that magnetometers, which are commonly embedded in smartphones and wearable devices, can serve as reliable and cost-effective cadence sensors without requiring additional hardware.

Recent advances have also focused on integrating sensing capabilities into more user-friendly form factors. Nijs et al. [28] demonstrated that inertial data collected from instrumented wireless earbuds can accurately estimate both cadence and stance time, achieving validity comparable to traditional foot-mounted sensors. This work highlights the potential of ear-level IMUs for continuous, unobtrusive gait monitoring. Although these methods are useful, they require the user to wear a device on the body, which would also necessitate an additional display system if the goal is to use the cadence data for interactive applications such as video games.

New modalities have been introduced to enable contactless or vision-based cadence estimation. Xuan et al. [12] presented CaNRun, an acoustic-based system that utilizes a smartphone’s built-in microphones to estimate running cadence on treadmills. Their method employs non-contact acoustic sensing and signal periodicity analysis to infer step frequency without requiring the user to wear any device, representing a significant step toward minimally intrusive cadence monitoring. However, the presence of multiple treadmills, such as in a gym environment, may introduce acoustic interference and reduce data accuracy. Complementarily, Marin-Lora et al. [15] developed a face-tracking method that applies computer vision algorithms to estimate cadence from RGB video streams recorded during treadmill running, achieving promising results in controlled indoor environments. This approach is particularly suitable for interactive applications such as gaming; however, the validation windows used in their experiments were 16 s long, which may cause a temporal mismatch between the runner’s real cadence and the on-screen visual feedback.

The paper by Roig-Maimó [29] introduced a camera-based head-tracking interface validated only in static or low-motion scenarios, requiring users to remain seated or hold the device steadily. Similarly, [30] proposed a multimodal human–computer interaction system for smart learning environments, leveraging facial and gesture recognition via front-facing cameras. While effective for controlled, sedentary use cases such as education, both approaches are constrained in dynamic contexts. In contrast, our system is specifically designed for fast-paced, interactive video games played while running on a treadmill. It distinguishes intentional lateral gestures from vertical oscillations, and includes FFT-based cadence estimation, enabling precise synchronization between the avatar and the user’s cadence.

The concept of avatar cadence is particularly important, as it directly influences users’ perception of exercise intensity and motivation. Studies have shown that virtual avatars can significantly impact user cadence and the overall exercise experience, reinforcing the necessity of integrating avatar interaction into treadmill systems [24,25].

In addition to visual feedback, prior research has explored different smartphone-based cadence estimation methods. Karei et al. [11] used the smartphone’s accelerometer, but this required mounting the device on the treadmill, limiting usability. Xuan et al. [12] proposed an acoustic-based cadence estimation using the smartphone’s microphone, avoiding wearables but introducing sensitivity to ambient noise typical in gyms. Marin-Lora et al. [15] developed a facial tracking method leveraging oscillatory facial movement during running; however, it required processing long video segments of 512 frames (about 16 s) to achieve less than 5% error, resulting in high computational costs and reduced responsiveness.

Although sophisticated global optimization algorithms, such as the memory-based hybrid crow search algorithm, have shown strong performance in solving constrained numerical problems [31], their design focuses on complex offline optimization rather than lightweight, real-time signal processing.

Marín-Lora et al. [7] also designed a treadmill game controlled by smartwatch-based gestures, later comparing smartwatch, accelerometer, and facial interactions [32]. While all methods provided technically viable gesture recognition and cadence estimation, users consistently rated facial tracking as the most comfortable and least intrusive option.

Table 1 provides an overview of interaction systems for running or motion detection based on body tracking, detailing their underlying technologies, strengths, and limitations. Most existing solutions rely on external hardware, complex setups, extended input windows, or are designed for static environments, whereas our system enables real-time, hands-free interaction using only the smartphone’s front-facing camera during treadmill running.

In light of these limitations, we propose a low-cost, portable solution that leverages facial tracking through a smartphone’s front-facing camera. By combining natural head gestures and real-time cadence estimation using short video windows, our system aims to enhance accessibility and responsiveness in treadmill-based interaction.

**Figure 1:**
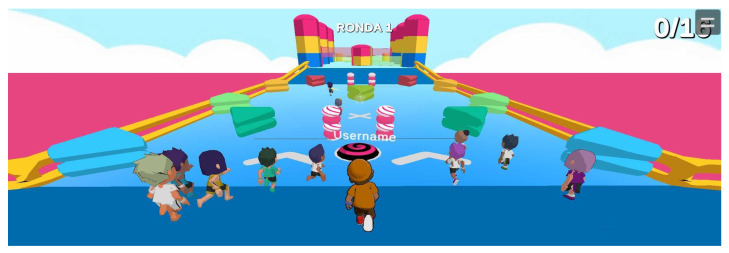
Screenshot showing the race environment.

Figure 1 shows the game interface as seen by the user during a treadmill session. The avatar’s movement responds in real time to facial tracking, providing immediate visual feedback to the runner.

Unlike previous face-tracking methods that required longer video sequences, our system offers a more responsive interaction by reducing the duration needed for accurate cadence estimation. By processing facial motion through FFT analysis, it enables real-time feedback without relying on external sensors or complex setups. This improves system accessibility while addressing latency issues observed in earlier approaches.

## 3. System Design and Implementation

**Figure 2:**
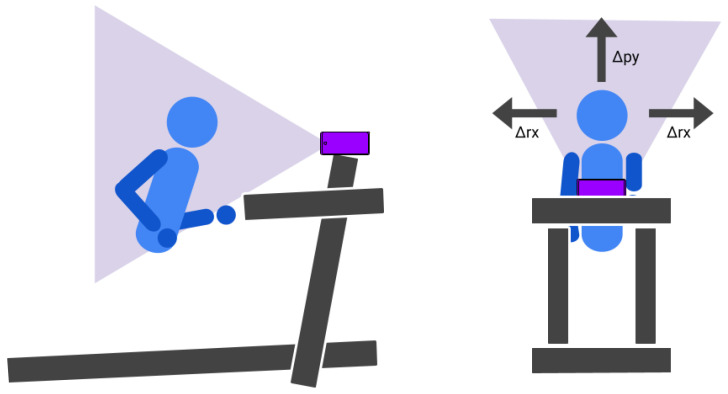
Diagram of the lateral and frontal configuration of the system with the treadmill and the smartphone pointing at the runner’s face to detect variations in horizontal rotation and vertical position.

To ensure the effective operation of the proposed interaction system, the setup requires only a treadmill and a smartphone. The smartphone must be positioned such that it simultaneously displays the game and captures the user’s face using the front-facing camera. This configuration enables the estimation of both cadence and movement direction in real time. Figure 2 illustrates this setup, showing the lateral and frontal placement of the smartphone in relation to the treadmill and the user.

The avatar’s in-game speed and direction are continuously adjusted based on the user’s cadence and lateral head movements. For an enhanced immersive experience, the game can be mirrored onto a Smart TV placed in front of the treadmill, offering a larger and more engaging visual environment.

Importantly, the smartphone remains fixed on the treadmill, ensuring stable capture of the user’s face. Head movements to the left or right are interpreted as direction changes, while the avatar’s running cadence is synchronized with the estimated cadence of the user. This configuration eliminates the need for additional devices such as smartwatches or motion sensors, offering a simplified and user-friendly solution [7,15].

### 3.1. Facial Detection-Based Interaction System

The interaction system was designed to offer an intuitive, immersive gameplay experience through real-time facial tracking, eliminating the need for handheld or wearable devices. The game responds to natural head movements by controlling both avatar direction and cadence.

The implementation was carried out in Unity (Unity Technologies, San Francisco, CA, USA) using the AR Foundation framework, which integrates ARKit (iOS) and ARCore (Android) to access device camera feeds and enable facial detection capabilities. Unity was selected due to its robust cross-platform AR tools and seamless support for real-time computer vision applications [20,29].

**Figure 3:**
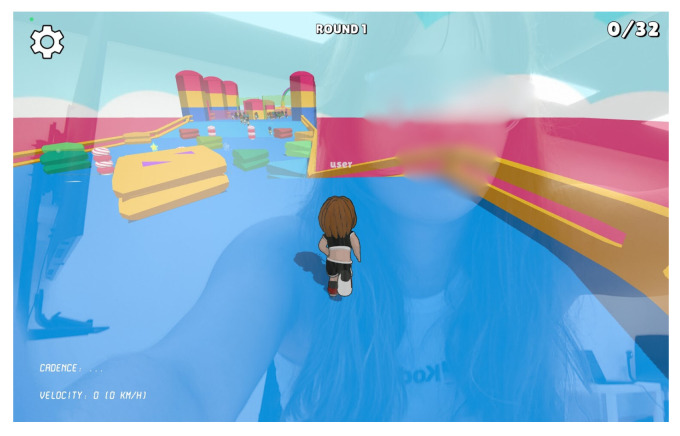
On-screen feedback shown when facial tracking is lost. The smartphone displays a semi-transparent camera feed to help users realign themselves in front of the sensor.

The interaction design consists of a three-stage process:

1. Facial Detection and Tracking: The camera continuously analyzes frames to detect facial landmarks using point-tracking algorithms. These landmarks provide spatial coordinates (x, y, z) and orientation angles (pitch, yaw, roll) of the user’s head.
2. Motion Mapping: The horizontal rotation of the head (yaw) is scaled by an empirically defined factor and mapped to the avatar’s directional movement. Smooth transitions are achieved through interpolation, minimizing abrupt changes and ensuring user comfort. The logic is described in Algorithm 1, which operates as follows: First, the algorithm retrieves the current yaw angle of the user’s head, representing horizontal rotation relative to the camera. This angle is multiplied by a rotation intensity factor to adjust sensitivity. Next, the scaled angle is clamped within a predefined range (−90^o^ to 90^o^) to prevent extreme or abrupt avatar rotations. The clamped angle is then converted into a quaternion representing the target rotation in 3D space. Finally, the avatar’s current rotation is smoothly interpolated towards this target using spherical linear interpolation (Slerp) with a smoothing factor, ensuring gradual and natural directional transitions that enhance user comfort during treadmill gameplay.
  Although no user-specific calibration is required, the rotation intensity and smoothing factor used in the directional control were empirically tuned during preliminary testing to optimize responsiveness and stability during gameplay.
3. Feedback Mechanism: If facial tracking is lost, the smartphone displays the camera feed with 50% opacity overlayed on the game view. This visual cue allows users to adjust their position and reestablish tracking without interrupting the gameplay session  [22,33]. Figure 3 shows the on-screen interface used to guide the user back into position. This passive visual feedback enhances usability and minimizes interaction disruption during gameplay.

**Algorithm 1** Avatar Rotation Based on Head Orientation1:angle ← GetYawAngle(faceTransform)2:rotationWithMult ← angle × rotationIntensity3:clampedRotation ← Clamp(rotationWithMult, $-90^{\circ }$, $90^{\circ }$)4:desiredRotation ← Quaternion.Euler(0, clampedRotation, 0)5:avatar.rotation ← Slerp(avatar.rotation, desiredRotation, smoothingFactor)

The use of face-based control for interaction aligns with recent research in facial gesture interfaces and mobile gaming, which emphasize hands-free accessibility and intuitive control [34,35]. However, our implementation distinguishes itself by achieving robust performance using only smartphone hardware, addressing limitations found in systems that depend on specialized devices such as webcams or HMDs [17,19].

The system achieves real-time capability through a combination of design and implementation decisions. The avatar’s direction, based on horizontal head rotation, is updated every frame, ensuring an immediate and fluid response to the user’s lateral movements through smooth interpolation. Furthermore, frame rate estimation entails an average processing time of 1 ms per FFT calculation, which is negligible compared to the update interval and ensures immediate availability of the new frame rate. All processing is executed on the main Unity thread, avoiding additional latency due to concurrency. Furthermore, the system was tested on mid-range smartphones capable of maintaining a constant refresh rate of 30 fps, which allows the system to be classified as interactive in real time.

### 3.2. Cadence Estimation Method

To estimate the runner’s cadence in real time, the vertical position of the nose reference point was analyzed using the XR Face Tracking plugin in Unity. The data were sampled at 30 Hz, corresponding to the capture rate of the smartphone’s front-facing camera. The choice of a 128-frame window was informed by preliminary testing in which different window sizes, ranging from 64 to 256 frames, were evaluated. Shorter windows (e.g., 64 frames) produced faster updates but resulted in increased noise sensitivity and less stable peak detection. Conversely, longer windows (e.g., 256 frames) improved frequency resolution but introduced perceptible delays that negatively affected responsiveness. The 128-frame window offered a practical compromise: it ensured sufficient spectral resolution for robust cadence estimation while maintaining a short update interval (approximately 4.27 s), making it suitable for real-time treadmill interaction. For each estimation, this 128-sample window was collected without filtering, as preliminary tests showed that preprocessing was unnecessary. These samples were transformed into complex numbers and processed using a radix-2 Fast Fourier Transform (FFT) implementation in Unity. The resulting frequency spectrum contains one complex value per bin, from which the magnitude of each frequency component was computed as the modulus of the complex number. To avoid the dominant low-frequency component caused by slow head displacement, the first five bins were discarded.

The algorithm then searched for the bin with the maximum magnitude in the positive frequency range. This index, maxFFTIndex, was mapped to its corresponding frequency (in Hz) using the duration of the sampling window:

$$\mathrm{frequency}\;\left(\mathrm{Hz}\right)=\frac{\mathrm{maxFFTIndex}}{128/30}$$

The dominant frequency represents the runner’s cadence in steps per second, which was then multiplied by 60 to express cadence in steps per minute. This value was used to control both the avatar’s animation playback speed and its forward movement in the game environment.

Algorithm 2 computes the runner’s cadence from vertical nose motion using a radix-2 FFT on a 128-sample window, discarding the first five bins, selects the dominant frequency, and converts it to steps per minute.
**Algorithm 2** Cadence Estimation and Avatar Speed Update1:**Input:** FFTdata[128]2:**Output:** avatar.speed3:avatar.speed ← EstimarCadenciaFFT(FFTdata)

Algorithm 3 estimates the user’s running cadence based on vertical nose motion captured by the smartphone’s front camera. Data are collected in 128-sample windows at 30 FPS, converted to complex numbers, and processed using a radix-2 FFT. To reduce the influence of the low-frequency peak caused by slow head displacement, the first five bins are discarded. The bin with maximum magnitude in the positive frequency range is selected as the dominant frequency, which is converted to steps per minute. During testing, raw cadence estimates obtained via FFT were observed to slightly overestimate the actual cadence, particularly at lower running speeds. To address this, a simple two-parameter linear correction model (ordinary least squares), fitted using video-based ground truth data, was applied to the estimated frequency before conversion. This adjustment refines only the global mapping between frequency and cadence, does not require individual calibration, and ensures accurate and reliable cadence estimation across participants and running speeds, as it depends on the temporal pattern of facial movement rather than absolute geometry.
**Algorithm 3** EstimarCadenciaFFT1:**function** EstimarCadenciaFFT(FFTdata[128])2:    **for** i = 0 **to** FFTdata.Length-1 **do**3:        complexData[i] ← Complejo(FFTdata[i], 0)4:    **end for**5:    FFT(complexData)                             ▹ Radix-2 FFT6:    sampleRate ← FFTdata.Length / 307:    maxMagnitude ← 08:    **for** i = 5 **to** complexData.Length/2 **do**9:        **if** complexData[i].Magnitude > maxMagnitude **then**10:           maxMagnitude ← complexData[i].Magnitude11:           maxFFTIndex ← i12:        **end if**13:    **end for**14:    frequencyHz ← maxFFTIndex / sampleRate15:    stepsPerMinute ← frequencyHz × 6016:    **return** stepsPerMinute17:**end function**

**Figure 4:**
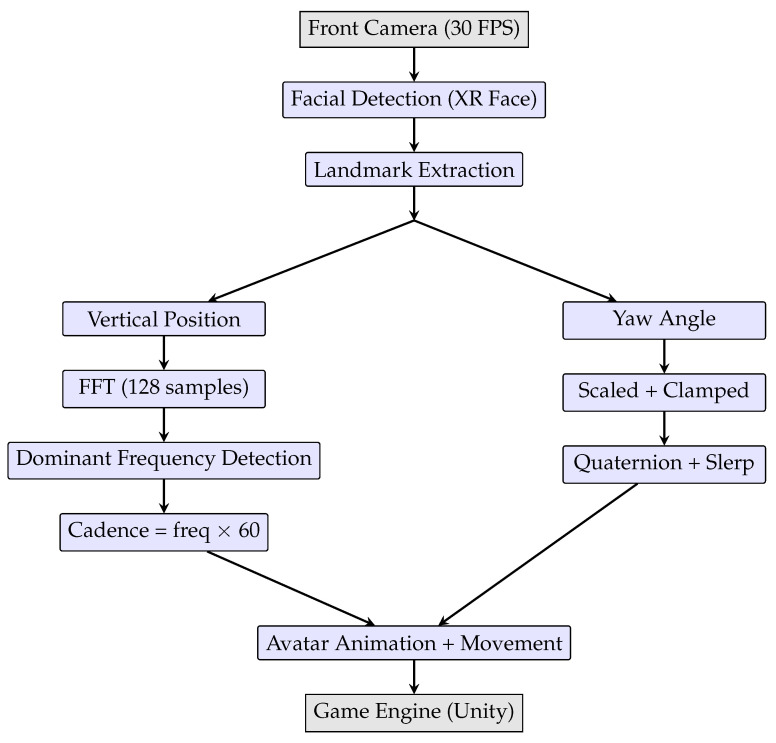
System architecture and data-processing pipeline. Facial landmarks are extracted from the front camera input and split into two branches: yaw angles for directional control, and vertical motion for cadence estimation via FFT. Both influence avatar control in the game engine.

A complete visual summary of the proposed system architecture and data-processing pipeline is presented in Figure 4, integrating the facial tracking, signal analysis, and avatar control components described above.

## 4. Experimental Setup and Data Processing

This section details the procedures used to evaluate the proposed interaction system. It includes the experimental design, participant information, recording setup, and offline data-processing methods used to validate the accuracy of cadence estimation via facial tracking.

### 4.1. Participant Study and Equipment Setup

To validate the real-time cadence estimation method in a treadmill video game context, we conducted an experimental study involving treadmill running activities.

The study involved 11 participants (5 women and 6 men) with ages ranging from 23 to 57 years (mean = 28.5, SD = 9.1). To characterize the sample, two self-reported background variables were collected using 5-point Likert scales ranging from 0 (lowest) to 4 (highest). In terms of fitness level, the average score was 1.73 (SD = 0.79), indicating generally low-to-moderate physical activity; 64% of participants rated themselves between 1 and 2, while one participant reported a sedentary lifestyle, and two indicated a moderate-to-high activity level. For gaming experience, the mean score was 2.18 (SD = 0.98), suggesting moderate familiarity with video games; 73% of participants reported a score of 2 or higher, and only one participant reported no prior gaming experience. All participants provided informed consent and reported no physical limitations that would interfere with treadmill use. Each session lasted approximately 20 min and was conducted individually. The study received ethical approval from the institution’s Ethics Committee.

The experimental setup included a BH-RC09 treadmill (BH Fitness, Vitoria-Gasteiz, Spain) and two Redmi Note 8 smartphones (64 GB storage, 4 GB RAM; Xiaomi Corporation, Beijing, China), both equipped with front cameras (13 MP, f/2.0 aperture, 29 mm field of view) operating at 30 frames per second. The treadmill’s 155 cm × 55 cm running surface allowed for slight lateral movements during gameplay, making it suitable for inexperienced users.

All recordings were conducted under standard indoor conditions (≈24 ºC), with the treadmill incline fixed at 0% throughout all sessions. The smartphone running the video game was rigidly mounted on the treadmill’s front frame to ensure consistent frontal capture of the participant’s face and maintain an approximate 50 cm participant-to-camera distance. The game interface, with head-tracking control for direction and cadence, was displayed on the smartphone screen, while a second smartphone simultaneously recorded the full activity for ground truth analysis.

Each participant completed three one-minute runs at fixed treadmill speeds of 6 km/h, 8 km/h, and 10 km/h. The facial motion was captured by the game system, while an external video recording served as reference for later step counting.

**Figure 5:**
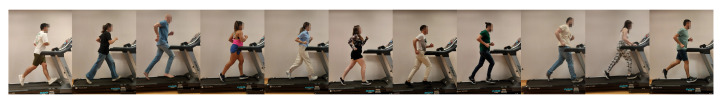
Sample frames extracted from video recordings during the experimental sessions.

Figure 5 shows example frames from the recorded videos used for offline analysis.

### 4.2. Ground Truth Acquisition and Signal Processing

The cadence estimation method based on vertical head movement and FFT, as described in Section 3, was evaluated offline. During each one-minute session, the Unity XR Face Tracking plugin recorded vertical face position data, which were exported in CSV format for analysis.

For each trial, a fixed 128-frame window was extracted from the central portion of the recording. This segment captured multiple steps while avoiding start-up or fatigue artifacts. To standardize analysis across all conditions, the first 10 s of each video were discarded, and the window was selected from the remaining 41.6 s of data.

Cadence was estimated using the FFT-based algorithm described previously. To correct the slight systematic overestimation observed, a simple linear regression model was trained using ground truth values. The resulting model, $\hat{y}=0.83x+13.70$, yielded a high coefficient of determination ($R^{2}=0.89$) and a standard error of 6.76 steps/min, indicating strong agreement. This corrected method is referred to as FFT-Corrected. A 5-fold cross-validation of the correction model resulted in a global mean absolute error (MAE) of 5.17 steps/min, supporting its robustness and generalization capability.

The ground truth for each run was obtained by manually counting the steps from the external video recordings. A Python script was developed using the MediaPipe (Google LLC, Mountain View, CA, USA) library [36] to detect body landmarks such as toes, heels, ankles, shoulders, and ears. Among these, the ear landmark proved to be the most stable for cadence detection and was used as the primary feature, validated through visual inspection.

For benchmarking purposes, a previously implemented method based on peak detection from the vertical displacement of the nose was also evaluated using 128-frame intervals. The estimation error of this method was compared with the FFT-Corrected results and the ground truth.

Hardware and Software. All data-processing tasks were performed on an OMEN HP 40L Gaming Desktop GT21-2xxx equipped with an Intel Core i7-14700K processor (28 CPUs, 3.4 GHz), 32 GB RAM, and an NVIDIA GeForce RTX 4070 Ti SUPER graphics card (NVIDIA Corporation, Santa Clara, CA, USA). The Unity application was developed using version 2021.3.25f1 under DirectX 11. This setup ensured optimal performance during both real-time capture and offline analysis.

## 5. Results and Evaluation

This section presents the performance evaluation of the proposed cadence estimation method based on Fast Fourier Transform (FFT-Corrected), and compares it against a conventional peak detection approach (Peaks) [15]. The actual cadence, extracted from video recordings using MediaPipe, serves as ground truth. Evaluation is performed using standard metrics, participant-level analysis, and agreement plots.

### 5.1. Estimation Accuracy and Method Comparison

To assess the accuracy of both methods, two standard error metrics were used: Root Mean Square Error (RMSE) and Mean Absolute Error (MAE) [37]. These were computed across all participants and treadmill speeds (6 km/h, 8 km/h, and 10 km/h).

Table 2 reports the average estimated cadence for each participant at the three treadmill speeds, comparing the FFT-Corrected and Peaks methods to the video-based ground truth. It shows that the FFT-Corrected method consistently approximated the ground truth across all participants and speeds. At most, estimated values remained within ±5 steps/min of the video reference. In contrast, the Peaks method exhibited large deviations in several cases. These results illustrate the greater stability of FFT-Corrected, which maintained a narrower distribution and fewer extreme errors regardless of running speed.

Table 3 summarizes the RMSE and MAE obtained by both methods at each speed. The FFT-Corrected method achieved consistently low errors, with a total RMSE of 6.74 and MAE of 5.16 steps/min, corresponding to relative errors below 5%. By contrast, the Peaks method showed significantly higher estimation errors, with a total RMSE of 35.16 and MAE of 26.28 steps/min. The performance was notably poorer at 8 km/h, where the RMSE exceeded 40 steps/min, suggesting that this method lacks the robustness necessary for short time-window cadence estimation.

Given that the average cadence ranges from 132 to 168 steps per minute, an estimation error of approximately 8 steps per minute is practically imperceptible in the avatar’s movement, resulting in a smooth gaming experience.

To statistically validate the difference in estimation accuracy between methods, a Wilcoxon signed-rank test was applied to the individual MAE and RMSE values across all participants. The results indicated that the FFT-Corrected method significantly outperformed the Peaks method, both in terms of MAE (Z = −5.012, *p* < 0.001) and RMSE (Z = −5.012, p<0.001). The effect size was large in both cases (r = −0.873), indicating a strong practical relevance of the improvement. In all cases, estimation errors were lower with the FFT-Corrected method, confirming the robustness and consistency of the proposed approach.

### 5.2. Inter-Participant Variability

To evaluate the consistency of each method across individuals, Table 4 presents the RMSE per participant and speed. The FFT-Corrected method exhibited stable performance, while the Peaks method was more variable and error-prone, with extreme deviations such as 79.23 steps/min for Participant 6 at 8 km/h.

Table 4 further illustrates the per-participant variability. The FFT-Corrected method yielded low and consistent RMSE values, with most participants remaining below 10 steps/min across all treadmill speeds. In contrast, the Peaks method exhibited high variability, with several participants (such as Participants 6 and 10) reaching RMSE values above 30 steps/min. This variability suggests a lack of robustness in the Peaks method when the time window is short.

To assess the magnitude of the observed effect, Cohen’s d was calculated, resulting in a value of −2.075, which indicates a very large effect and consistent superiority of the FFT-Corrected method.

### 5.3. Agreement with Ground Truth

Agreement between estimated and actual cadence values was further assessed using Bland–Altman plots. These illustrate the difference versus the mean of the two measures, providing insight into systematic bias and limits of agreement.

**Figure 6:**
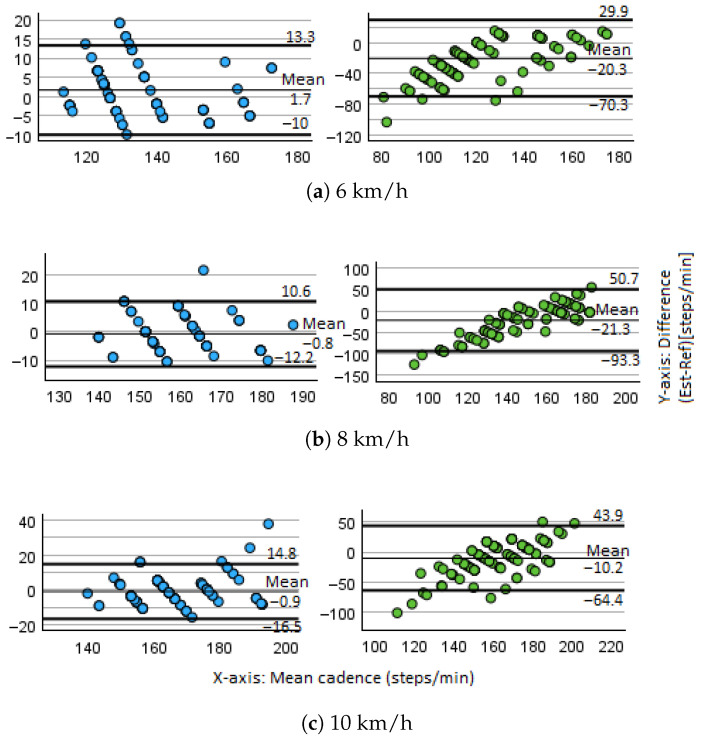
Bland–Altman plots comparing FFT-Corrected (left) and Peaks (right) cadence estimation against ground truth for three treadmill speeds. Each point represents the difference between the estimated and actual cadence values plotted against their mean. The horizontal axis shows the average cadence between the two methods, while the vertical axis shows the difference between the estimated and ground truth values. The plots include the mean bias and the limits of agreement (±1.96 SD).

Figure 6 displays the Bland–Altman plots for the three treadmill speeds. The FFT-Corrected method (left) shows narrow and consistent agreement, while the Peaks method (right) exhibits wider and erratic deviations.

Table 5 summarizes the Bland–Altman statistics for both estimation methods across the three treadmill speeds. The FFT-corrected method demonstrated minimal bias and narrow limits of agreement across all speeds. At 6 km/h, the mean difference was 1.65 steps/min, with limits of agreement (LoA) of ±11.69 steps/min. At 8 km/h, the bias decreased to −0.79, with LoA of ±11.43 steps/min, and at 10 km/h, the bias reached −0.85 steps/min with LoA of ±15.65 steps/min. These narrow LoA intervals demonstrate a consistent level of precision and stability across all running speeds.

In contrast, the Derivative-based method exhibited substantially poorer agreement. At 6 km/h, the bias was large and negative (−20.28 steps/min), with very wide LoA of ±50.05 steps/min. This pattern became even more pronounced at 8 km/h, where the bias was −21.33 steps/min and the LoA expanded to ±71.99 steps/min. Although the bias decreased slightly at 10 km/h (−10.24 steps/min), the LoA remained extremely broad (±54.11 steps/min). These results reveal the presence of systematic underestimation and large variability in the Derivative method. This high variability compromises the suitability of the Peaks method for short time-window estimation, particularly in real-time interactive applications.

Correlation and reliability metrics further supported these findings, as can be seen in Table 6. The FFT-Corrected method showed an excellent linear and monotonic association with the video-based cadence, with a Pearson correlation of r=0.941 (p<0.001) and a Spearman correlation of ρ=0.939 (p<0.001). Reliability indices were similarly strong: Cronbach’s α=0.969 and an intraclass correlation coefficient (ICC) of 0.940 (single measures) and 0.969 (average measures), with 95% confidence intervals ranging from 0.926 to 0.951, demonstrating excellent absolute agreement.

Conversely, the Derivative-based method yielded only moderate association with the ground truth (Pearson r=0.605; Spearman ρ=0.636; both p<0.001). Its reliability metrics reflected this reduced performance, with Cronbach’s α=0.663 and ICC values of 0.428 (single measures) and 0.600 (average measures), accompanied by substantially wider confidence intervals (0.217–0.580), indicating only fair agreement.

The Bland–Altman inspection revealed clear differences in the number and distribution of outliers between methods across all treadmill speeds. At 6 km/h, the Derivative-based method yielded three outliers below the lower limit of agreement, whereas the FFT-Corrected method showed six outliers above the upper LoA and none below, despite presenting substantially narrower LoA than the Derivative approach. At 8 km/h, the FFT-Corrected method exhibited one outlier above and none below, while the Derivative-based method presented one above and three below the LoA. At 10 km/h, the Derivative-based method showed two outliers above and five below, whereas the FFT-Corrected method displayed five outliers above and none below.

It is important to point out that the outliers of the FFT-Corrected method at higher speeds corresponded to participants running at high cadences, where the estimated cadence slightly exceeded the already elevated ground truth values, rather than reflecting unusually low reference cadences.

Although both methods produced atypical observations, the Derivative-based method demonstrated broader dispersion and a higher proportion of outliers falling below the LoA, indicating greater sensitivity to local signal irregularities. In contrast, outliers in the FFT-Corrected method were fewer at moderate speeds and remained within a narrower agreement range.

While prior work reported that derivative-based cadence estimation performed reliably when using 512-frame windows (≈16 s) [15], the present results show that, with a 128-frame window, the FFT-based approach maintains stable agreement and reduced dispersion. This supports its suitability for rapid update rates, enabling more responsive avatar synchronization during real-time treadmill interaction.

## 6. Conclusions

This work presented a real-time interaction system for treadmill-based video games, relying solely on facial tracking via a smartphone’s front-facing camera. The system enables running cadence estimation and directional control through natural head movements, without requiring any additional wearable devices or sensors.

The FFT-based method demonstrated robust and consistent cadence estimation across treadmill speeds, achieving a mean absolute error under 8 steps/min. This level of accuracy falls within the error ranges typically reported for cadence estimation using body-mounted accelerometers [11,28,38], while offering a non-invasive and more accessible solution.

Compared to a conventional peak detection approach, the FFT-corrected method demonstrated significantly better accuracy, lower bias, and more consistent performance across all speeds and participants. Its responsiveness with short time windows makes it especially suitable for real-time interactive applications.

In addition to its technical performance, the system offers practical advantages: simplified setup, intuitive directional control via head movements, and hands-free interaction. These features contribute to a more accessible and seamless user experience compared to traditional gesture-based systems.

## 7. Limitations and Future Work

While the proposed system demonstrated robust performance under controlled treadmill conditions, several limitations should be acknowledged.

First, the accuracy and stability of facial tracking may be influenced by suboptimal lighting, partial occlusions, and variability in user height or positioning relative to the camera. These factors can affect the quality of facial landmark detection and, consequently, the precision of cadence estimation.

Second, the study was conducted with a limited sample size of 11 participants. Although the results were consistent across individuals, the generalizability of the findings should be further evaluated through studies involving larger and more diverse populations, including different age groups and fitness levels.

Third, the current evaluation focused on short treadmill sessions at fixed speeds. Future work should explore system performance in longer trials, variable speed conditions, and free-paced running to better simulate real-world usage. As our evaluation relied on short, controlled trials, it did not allow us to assess potential long-term effects, such as tracking fatigue or the gradual degradation of landmark stability, during extended treadmill sessions. Therefore, future work will include longer continuous runs to examine system robustness over time. Furthermore, user-centered evaluations will be necessary to assess subjective dimensions such as engagement, usability, and cognitive load, and to compare the proposed system with widely used commercial exergames such as Zwift, Ring Fit Adventure, or Fitoon, focusing on performance, responsiveness, and user experience in real-life situations.

In addition, future work could explore adaptive mapping strategies to better relate head-oscillation frequency to actual cadence across different running styles and non-stationary rhythms. This may include user-specific calibration procedures as well as joint time–frequency analysis techniques (e.g., STFT or Wavelet Transform), as well as testing the system on a wider range of smartphone models from different device tiers.

To enhance adaptability, future iterations of the system may benefit from the integration of machine learning techniques for personalized cadence-speed mapping based on user profiles. Moreover, expanding the system to support other use cases (such as overground running or rehabilitation) could broaden its applicability. Longitudinal studies are also encouraged to assess the system’s potential impact on user motivation, adherence to exercise, and long-term health outcomes. Finally, future developments could explore the integration of this system with augmented reality environments to enrich visual feedback and improve immersion, potentially leading to more engaging and effective workouts.

Taken together, these limitations highlight meaningful avenues for future research aimed at improving the scalability, robustness, and inclusivity of facial tracking-based interaction systems for fitness and health-oriented applications.

## Figures and Tables

**Table 1:** Comparison of treadmill interaction systems.

Ref	Technology	Interaction	Strengths	Limitations
[6]	Smartphone + treadmill sensors	Cadence-based control via treadmill speed	Natural treadmill-driven interaction; simple physical input	Requires treadmill hardware integration; not adaptable to other setups
[7]	Smartwatch with motion sensors	Arm gesture control via wearable	Non-intrusive; enables hands-free gesture input	Lower precision than camera-based methods; smartwatch required
[17]	Webcam + motion tracking	Face and hand gesture input	Natural and contactless; low-cost hardware	Sensitive to lighting; user must stay in camera view
[18]	Webcam + facial recognition	Head movement and facial input	Hands-free and expressive interaction	Sensitive to tracking errors and lighting conditions
[19]	HMD with facial capture	Facial expression tracking	High-fidelity facial animation in VR	Requires VR hardware; not suitable for treadmill use
[20]	Camera-based facial tracking	Facial expression input	Expressive, contactless control	Requires stable lighting and accurate tracking
[21]	Smartphone motion sensors	Device tilt and movement input	Intuitive and mobile; no external hardware needed	Limited precision; must be handheld
[22]	Front camera+ motion sensors + touchscreen	Head gestures and facial proximity	Versatile and expressive input	Requires precise tracking; affected by lighting
[9]	Treadmill-integrated video tracking	Full-body movement input	Natural control without wearables	Sensitive to occlusion and lighting
[10]	Depth camera + tracking algorithms	Full-body depth-based tracking	Robust to lighting; contactless interaction	Requires depth camera; sensitive to occlusion
[11]	Smartphone accelerometer	Body-worn motion input	Robust cadence detection across placements	Requires device on body; not fully hands-free
[12]	Smartphone microphone	Acoustic cadence detection	Hands-free and easy to deploy	Affected by noise; treadmill-specific
[15]	Smartphone camera + face tracking	Vertical head motion input	Contactless and accessible cadence estimation	Sensitive to occlusions and head instability
[29]	Front camera + head tracking	Head movement input	Hands-free and mobile-friendly	Depends on lighting and camera positioning
[30]	Multimodal (voice, gesture, facial input)	Facial and gesture-based control	Natural and accessible interaction	High computational load; modality fusion errors
Our system	Smartphone camera	Vertical head motion input	No additional hardware; hands-free; real-time cadence estimation	Requires the user’s face to remain aligned with the camera

**Table 2:** Comparison of estimated cadence by FFT-Corrected (FFT) and Peaks vs. actual cadence (Video) at three treadmill speeds.

	6 km/h			8 km/h			10 km/h		
**Ps**	**FFT**	**Peaks**	**Video**	**FFT**	**Peaks**	**Video**	**FFT**	**Peaks**	**Video**
1	134.92	100.50	123.94	149.92	108.75	152.30	154.92	143.75	159.07
2	126.17	112.50	121.18	158.67	184.50	156.06	161.17	175.50	175.56
3	126.17	100.71	123.97	158.67	165.00	161.52	186.16	198.00	177.31
4	166.17	148.64	166.82	168.66	169.09	170.57	172.41	173.18	174.03
5	114.93	85.91	115.82	147.42	135.00	149.41	151.17	151.36	154.89
6	138.67	31.36	141.88	151.17	83.18	151.28	157.42	102.27	160.72
7	134.92	73.75	130.26	177.41	171.82	167.70	188.66	160.38	170.95
8	127.42	95.77	133.49	163.67	160.91	183.92	168.66	171.82	194.01
9	152.42	140.00	156.09	163.67	110.00	164.27	171.16	162.27	175.90
10	126.17	87.27	119.76	151.17	91.36	156.45	162.42	136.36	163.82
11	126.17	95.45	122.32	148.67	139.50	141.63	151.17	143.18	146.66

**Table 3:** Cadence estimation errors (RMSE and MAE) by speed and method.“Total” indicates the global error across all trials.

Method	Metric	6 km/h	8 km/h	10 km/h	Total
FFT-Corrected	RMSE	6.16	5.86	7.99	6.74
	MAE	4.93	4.55	6.01	5.16
Peaks	RMSE	32.52	42.33	29.33	35.16
	MAE	25.48	31.00	22.37	26.28

**Table 4:** RMSE per participant at each speed using FFT-Corrected and Peaks methods.

Participant	6 km/h		8 km/h		10 km/h	
	FFT	Peaks	FFT	Peaks	FFT	Peaks
1	13.09	30.69	3.65	55.79	6.68	21.05
2	2.45	10.80	6.03	14.05	15.58	24.67
3	5.50	17.90	9.94	31.71	5.37	22.55
4	4.74	34.41	5.51	9.38	9.50	16.87
5	3.98	35.63	4.07	24.34	3.77	17.50
6	3.58	8.28	1.57	79.23	7.08	62.31
7	5.04	31.67	4.34	11.62	4.93	9.88
8	5.58	61.36	6.80	19.67	6.72	40.07
9	5.82	23.64	3.57	57.64	7.58	23.72
10	7.00	35.16	5.56	67.11	1.89	27.57
11	4.39	34.15	8.44	9.38	10.08	20.06

**Table 5:** Bland–Altman statistics for FFT-Corrected and Derivative methods across treadmill speeds. Mean represents the bias (method—video). LoA: limits of agreement.

Speed	Method	Bias (Mean)	SD	LoA (Mean ± 1.96·SD)
6 km/h	FFT-Corrected	1.646	5.962	1.646±11.69
	Derivative	−20.277	25.536	−20.277±50.05
8 km/h	FFT-Corrected	−0.798	5.832	−0.798±11.43
	Derivative	−21.330	36.729	−21.330±71.99
10 km/h	FFT-Corrected	-0.846	7.985	−0.846±15.65
	Derivative	−10.239	27.609	−10.239±54.11

**Table 6:** Comparison of validity and reliability metrics for FFT-Corrected and Derivative-based cadence estimation methods.

Metric	FFT-Corrected	Derivative	CI Lower	CI Upper
Correlation with Video (Ground Truth)				
Pearson *r*	0.941	0.605	–	–
Spearman ρ	0.939	0.636	–	–
Internal Consistency				
Cronbach’s α	0.969	0.663	–	–
Intraclass Correlation Coefficient (ICC, absolute agreement)				
ICC (single measures)	0.940	0.428	0.926/0.217	0.951/0.580
ICC (average measures)	0.969	0.600	0.961/0.356	0.975/0.734

## Data Availability

The data presented in this study are available on request from the corresponding author due to due to privacy and ethical restrictions related to participant data.
